# A Three‐Year Longitudinal Study of Athlete Mental Health: A Cricket Case Study

**DOI:** 10.1111/sms.70125

**Published:** 2025-08-28

**Authors:** George Ely, Tim Woodman, Ross Roberts, Eleri Jones, Lynsey Williams, Nicholas Peirce

**Affiliations:** ^1^ Institute for the Psychology of Elite Performance Bangor University Bangor UK; ^2^ Professional Cricketers' Association London UK; ^3^ National Cricket Performance Centre England and Wales Cricket Board Loughborough UK; ^4^ National Centre for Sport and Exercise Medicine Loughborough University Loughborough UK

**Keywords:** anxiety, cricket, depression, longitudinal, maladaptive behaviors, mental health, sport, wellbeing

## Abstract

The majority of research on athlete mental health is cross‐sectional. The aim of this research was to investigate whether elements of athlete mental health differ between discrete periods of a sporting cycle. We measured depression, anxiety, alcohol misuse, problem gambling, and wellbeing of elite male cricketers during the preseason, mid‐season, and off‐season across three years. We used multiple imputation to account for missing data and conducted repeated‐measures analysis of variance for each of the mental health markers. Depression and alcohol misuse levels significantly changed across time, whilst anxiety and wellbeing levels remained stable. Depression levels were highest during the mid‐season but appeared to return to a baseline during the off‐season and preseason. Alcohol misuse levels were highest during the off‐season. Differences in mental health between discrete time points of a sporting cycle demonstrate the temporal component of athlete mental health. From a research perspective, the findings highlight the importance of conducting longitudinal mental health monitoring that accounts for multiple discrete periods within the athlete's sporting cycle. Practically, the resetting of depression levels during the off‐season and preseason illustrates the importance of time away from competition and thus serves as a warning for administrators who are considering adding fixtures and ultimately lengthening playing seasons.

Various mental health consensus statements [1, 2] and systematic reviews [3, 4] in sport have been published in the last decade. Collectively, they speak to a need for greater understanding of athlete mental health to effectively inform national governing body policy and to personalize athlete support. However, a significant limitation of much of the research that informs consensus statements on mental health in elite sport is the heavy reliance on the findings from cross‐sectional designs and a lack of longitudinal research [5].

This reliance on cross‐sectional designs is particularly problematic in elite sport because of the structure of seasons and the unique stressors that athletes face [6]. That is, designs that do not account for the possibility that mental health likely fluctuates within a season/cycle limit understanding of athlete mental health. Recently, longitudinal approaches have been used to assess mental health [7]; although the impact of the sporting cycle has been acknowledged as a potential contributing factor to changes in mental health [8], and recent research has identified a linear increase in depressive symptoms during the playing portion of a football season [9], no research has specifically investigated whether athlete mental health changes between discrete time points within the sporting cycle.

Cricket has historically been discussed as a sport with mental health challenges [10] but has received minimal empirical investigation regarding player mental health [11]. To demonstrate the potential mental health challenges in cricket, previous monitoring of South African cricketer mental health found that 59% of cricketers were experiencing anxiety/depression [12] and that 26% were engaging in adverse alcohol use [13]. Recently, investigations have gone beyond charting cricketer mental health, citing a number of mental health‐related challenges and risk factors [14, 15, 16]. Of note is the perceived decrease in wellbeing as the cricket season progresses [14], as well as the reported anxiety‐inducing aspects of being a cricketer, such as contract status, which may become more prevalent at certain periods within a cycle [15]. Importantly, domestic professional cricket is a sport with obvious discrete periods within a 12‐month cycle. Specifically, it includes a preseason that runs from approximately the start of January to the end of March, a playing season that is split into different formats and takes place between April and October, and an off‐season that runs from October until January. Consequently, the present study uses professional cricket to investigate whether core elements of mental health change in relation to a sporting cycle.

## 1 Materials and Methods

### 1.1 Study Design and Participants

Following institutional ethical approval (SSHES P02‐20/21), we collected data three times per year for three years (2021, 2022, and 2023). Specifically, we used online surveys to collect data regarding the preseason, in‐season, and off‐season of the England and Wales domestic cricket season. Preseason data were collected toward the end of the preseason period, in‐season data were collected at the midpoint of the playing season (late July) prior to players leaving for The Hundred (England and Wales's franchise‐based cricket tournament), and off‐season data were collected between 6 and 8 weeks after the playing season had ended (the date of this varied as teams finished the season at different times). The first page of the survey was the information sheet, followed by an informed consent statement. Participants were also required to state a preferred referral pathway (see Appendix S1 for more details regarding referral). Participants then completed a series of demographic questions regarding age, relationship status, contract status, ethnicity, level of play, length of time as a professional cricketer, and their role within a team (e.g., batter). After the demographics section, players completed the Patient Health Questionnaire (PHQ‐9 [17]), Generalized Anxiety Disorder Scale (GAD‐7 [18]), Alcohol Use Disorders Identification Test Consumption (AUDIT‐C [19]), Problem Gambling Severity Index (PGSI [20]), and World Health Organization Index (WHO‐5 [21]). There was an attention check question immediately after the PHQ‐9 and GAD‐7. Each survey followed the same format with, if applicable, changes to the stem of questionnaires to reflect the time period. For example, with the PGSI, instead of asking about gambling behaviors over the last 12 months we asked, “since the season began …” or, “since the end of the season …”.

Five hundred and two male cricketers (approximately 77% of the total population) from England and Wales aged 16–40 years old (M_age_ = 23.18 years, SD = 5.97) took part in the study. All participants came from the 18 first‐class cricketing counties in England and Wales. Regarding ethnicity, 77.2% were White, 7.5% were Asian/Asian British, 2.4% were Mixed/Mixed ethnic groups, 1% were Black/African/Caribbean/Black British, 1% regarded themselves as an “Other” ethnic group, and 10.9% did not report ethnicity. At their first data collection, participants regarded their playing level as either academy (*n* = 125), 2nd XI (*n* = 95), 1st XI (*n* = 262), or international (*n* = 20). The number of seasons players had been employed as professional cricketers ranged from 0 (academy players) to 20 (M_pro seasons_ = 5.31, SD = 5.61).

### 1.2 Thresholds

A PHQ‐9 score of 10 or more was used as a threshold to identify participants likely experiencing moderate levels of depression symptoms [22]. A GAD‐7 score of 8 or more was used to determine individuals likely to be experiencing some form of anxiety disorder [23]. The GAD‐7 has been identified as a metric capable of capturing anxiety symptoms related to PTSD and phobias in addition to generalized anxiety disorder. For the purpose of this research, we consider the GAD‐7 as a measure of trait anxiety (mental ill‐health). For males, a score of 4 or more on the AUDIT‐C indicated a likelihood of alcohol misuse [24]. We considered three thresholds for the PGSI. Specifically, scoring from 1 to 2 suggested an individual was a low‐risk candidate to develop a gambling problem; scoring 3–7 suggested an individual was a moderate risk candidate; and a score of 8 or more suggested an individual had a gambling problem [25]. Notably, the PHQ‐9, GAD‐7, AUDIT‐C, and PGSI are all recommended within the Sport Mental Health Assessment Tool 1 (SMHAT‐1 [26]).

### 1.3 Data Analyses and Statistics

We calculated means, standard deviations, and the number of individuals meeting diagnostic thresholds via SPSS. When reporting the number of individuals meeting diagnostic thresholds, we did not use multiple imputation (MI) so as to avoid ambiguity regarding the number of people meeting clinical thresholds for a mental health problem. Consequently, sample sizes vary for each time point. To make comparisons between and within sporting cycles, we used repeated measures analysis of variance (ANOVA). We chose to use multiple ANOVAs rather than multivariate analysis of variance (MANOVA) because we were interested in the specific changes, or lack of, for each variable in isolation, as opposed to whether some sort of combination of the variables changes across time [27]. Although we acknowledge that there is likely some statistical overlap between our measures of interest, they retain enough conceptual uniqueness to warrant investigation in their own right. We used G*Power 3.1.9.7 [28] and a small effect size of 0.2 [29] to determine the appropriate sample size. For 1 × 9 repeated measures ANOVA, *α* of 0.05, 1 − *β* of 0.95, and an estimated correlation among repeated measures of 0.5, the minimum sample size needed to detect a significant effect was 33.

71.95% of the data were regarded as missing. This was mostly because of individuals who had only completed one data collection. Missing data were assumed to be missing completely at random (MCAR), confirmed by a nonsignificant Little [30] MCAR test (chi‐square = 2768.01, df = 3121, *p* = 1.00). MI can be used to impute data in instances where the participant has not completed a data collection [31]. Given the temporal component of the research, we did not deem it appropriate to impute data for individuals who only had data for one time point. Instead, we used MI to enable us to conduct repeated measures ANOVA using data from individuals who had completed at least four of the nine time points (*n* = 115). The proportion of missing data for this subset was 42.5%. The data remained MCAR (chi‐square = 1707.77, df = 1768, *p* > 0.05). We conducted repeated measures ANOVAs and MI using R with the *mice* [32], *miceadds* [33], *lme4* [34], and *mitml* [35] packages. We used the “pmm” method via the *miceadds* package on R to impute data using a linear mixed model with predictive mean matching (PMM). With this method, to account for the nested nature of the repeated measurements, the variables used within the MI to predict the missing data are the variables in question at different time points as opposed to auxiliary variables such as demographics. To prevent power fall‐off, we performed 100 imputations with 20 iterations per imputation [36]. Results from analysis of each imputation were pooled using the moment‐based statistics method (*D*_1_) [37], which is an extension of Rubin's rules [38]. Example R code for the repeated measures ANOVA can be found in Appendix S2.

## 2 Results

Aligned with previous recommendations [39], where possible, we have considered mental health markers from both a categorical and continuous perspective. By categorizing individuals as meeting a threshold or not, we are able to determine the number of individuals likely to be experiencing a specific form of mental ill‐health, while considering the mental health marker as a continuous variable acknowledges mental ill‐health and maladaptive behaviors at subclinical levels [2] and mitigates against Type II error [39]. We have reported the number of individuals meeting predetermined thresholds of mental health markers in Table 1 and reported population means in Table 2.

**TABLE 1:** Number of players meeting clinical threshold.

Mental health measure	Threshold	Data collection								
		Pre 1	Mid 1	Off 1	Pre 2	Mid 2	Off 2	Pre 3	Mid 3	Off 3
		Sample size								
		245	144	129	166	117	106	153	103	111
PHQ‐9	10	4 (1.6%)	5 (3.5%)	3 (2.3%)	1 (0.6%)	5 (4.3%)	2 (1.9%)	3 (2%)	5 (4.9%)	3 (2.7%)
GAD‐7	8	10 (4.1%)	6 (4.2%)	0	6 (3.6%)	10 (8.5%)	5 (4.7%)	2 (1.3%)	2 (1.9%)	2 (1.8%)
PGSI	1–2	25 (10.2%)	1 (0.7%)	9 (7%)	6 (3.6%)	3 (2.6%)	6 (5.7%)	7 (4.6%)	3 (2.9%)	5 (4.5%)
	3–7	3 (1.2%)	1 (0.7%)	4 (3.1%)	3 (2.6%)	1 (0.8%)	0	3 (2%)	1 (0.9%)	3 (2.7%)
	8+	2 (0.8%)	0	0	0	0	0	0	0	0
AUDIT‐C	4–9	114 (46.5%)	70 (48.6%)	86 (66.7%)	104 (62.7%)	72 (61.5%)	68 (64.2%)	94 (61.4%)	57 (55.3%)	76 (68.5%)
	10+	0	0	3 (2.3%)	0	0	0	0	0	0

*Note:* Sample size varies between data collections. A score of 10 or more on the Patient Health Questionnaire (PHQ‐9) indicates a person likely to be experiencing moderate levels of depression. A score of 8 or more on the Generalized Anxiety Disorder Scale (GAD‐7) indicates a person likely to be experiencing some form of anxiety disorder. A score of 1 or 2 on the Problem Gambling Severity Index (PGSI) indicates someone is a low‐risk problem gambler; a score of 3 or more indicates the person is a high‐risk problem gambler. A score of 4 or more for men on the Alcohol Use Disorders Identification Test Consumption (AUDIT‐C) indicates a likelihood of alcohol misuse, while a score of 10 or more suggests the person is an alcoholic. Percentages are included to aid comparison between time points; however, this should be done with caution as participants in each data collection vary.

Abbreviations: 1, year 1 (2021); 2, year 2 (2022); 3, year 3 (2023); Mid, mid‐season; Off, off‐season; Pre, preseason.

**TABLE 2:** Mean scores for the mental health markers analyzed using repeated measures ANOVA.

Mental health measure		Data collection								
		Pre 1	Mid 1	Off 1	Pre 2	Mid 2	Off 2	Pre 3	Mid 3	Off 3
PHQ‐9	Mean	1.57	2.18	2.07	1.70	3.40	2.27	1.60	2.76	2.01
	Standard deviation	2.03	2.57	2.67	2.02	3.81	3.04	2.03	3.50	2.63
GAD‐7	Mean	1.66	1.71	1.94	1.86	2.61	2.54	1.65	2.48	2.57
	Standard deviation	2.27	2.70	2.29	2.42	3.05	3.57	2.06	3.35	3.38
AUDIT‐C	Mean	3.46	3.39	4.79	3.77	4.15	4.48	3.85	3.66	4.11
	Standard deviation	2.56	2.28	2.89	2.55	2.41	2.88	2.55	3.19	3.61
WHO‐5	Mean	18.58	17.75	18.08	18.44	16.68	18.13	18.70	17.51	18.33
	Standard deviation	3.28	4.57	4.43	3.18	4.88	5.05	4.19	6.02	5.59

*Note:* The sample here is the subset who completed 4 or more data collections (*n* = 115). The *mice* package on R was used to calculate means and standard deviations utilizing multiple imputation.

Abbreviations: Mid, mid‐season; Off, off‐season; Pre, preseason.

We conducted repeated measures ANOVAs for depression, anxiety, alcohol, and wellbeing across all nine time points using the subset of individuals who had completed four or more surveys (*n* = 115). It was not possible to conduct a repeated measures ANOVA for problem gambling due to the small number of people endorsing any item on the PGSI. The means and standard deviations for the mental health markers analyzed are presented in Table 2.

**FIGURE 1:**
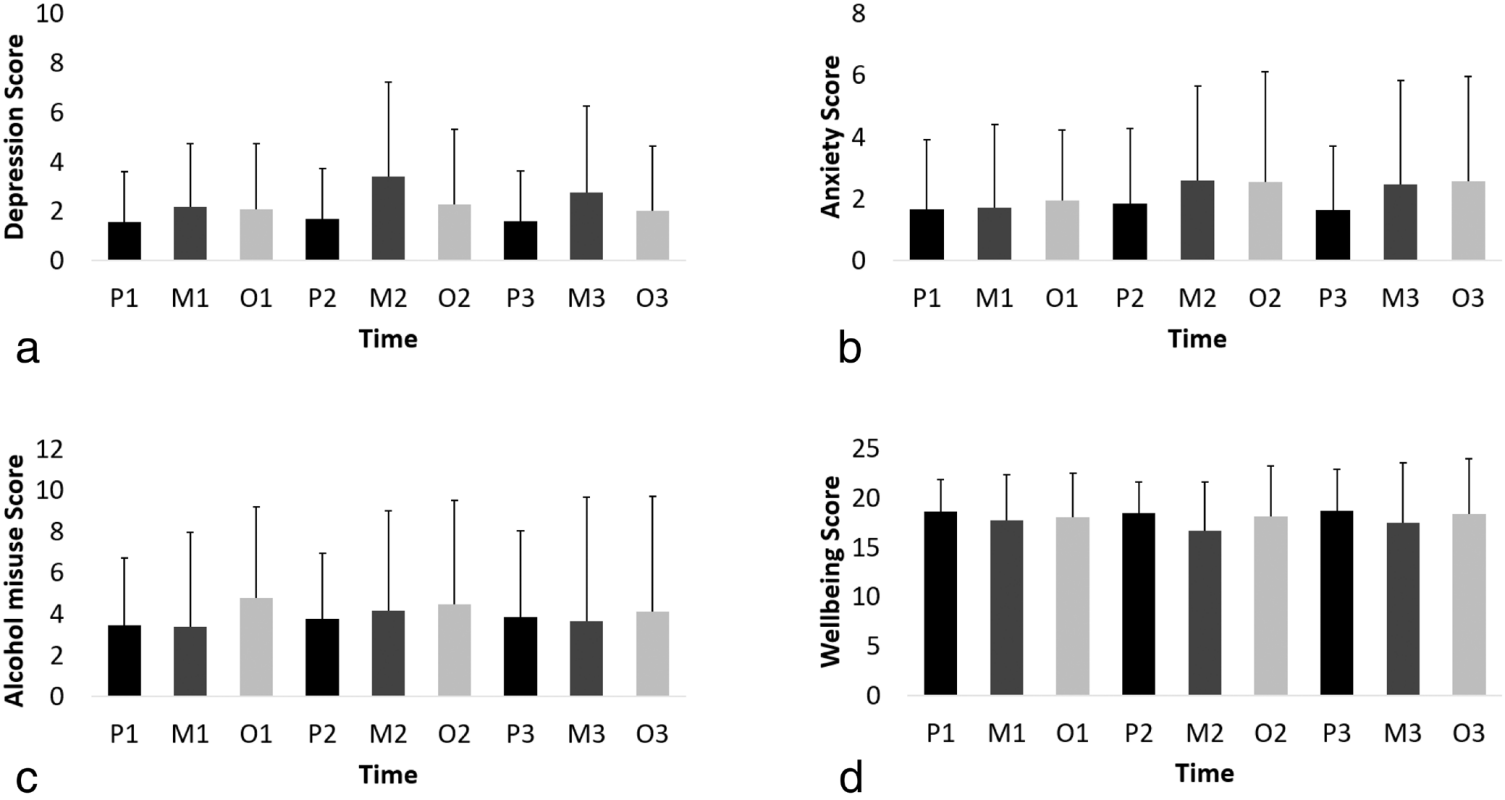
Means and standard deviations for the subset of individuals included in repeated measures ANOVA. (a) Depicts depression scores over time. (b) Depicts anxiety scores over time. (c) Depicts alcohol misuse scores over time. (d) Depicts wellbeing scores over time. M, mid‐season; O, off‐season; P, preseason; 1, year 1 (2021); 2, year 2 (2022); 3, year 3 (2023). Bar indicates mean, error bar indicates standard deviation.

A repeated measures ANOVA revealed a significant difference in depression scores across time, *F*(8, 8685.41) = 2.61, *p* < 0.01. Tukey's post hoc pairwise comparisons revealed that depression scores at mid‐season 2 (*M* = 3.40) were significantly higher than all other depression scores, with Cohen's *d*'s ranging between *d* = 0.48 and *d* = 0.78, except at mid‐season 3 (*M* = 2.76). Depression scores at mid‐season 3 (*M* = 2.76, *d* = 0.56) were also significantly higher than depression scores at preseason 1 (*M* = 1.57; see Figure 1a). A repeated measures ANOVA revealed a significant difference in alcohol misuse scores across time, *F*(8, 7886.24 = 2.60, *p* < 0.01). Tukey's post hoc pairwise comparisons indicated that alcohol misuse scores for off‐season 1 (*M* = 4.79) were significantly higher than all preseason alcohol misuse scores, ranging between *d* = 0.42 and *d* = 0.54, and both the mid‐season 1 (*M* = 3.39, *d* = 0.61) and mid‐season 3 (*M* = 3.66, *d* = 0.48) alcohol misuse scores. The off‐season 1 mean score difference with mid‐season 2 (*M* = 4.15) approached conventional statistical significance (*p* = 0.07, *d* = 0.28). Alcohol misuse scores were also significantly higher in off‐season 2 (*M* = 4.48) than they were in preseason 1 (*M* = 3.46, *d* = 0.48) and preseason 2 (*M* = 3.77, *d* = 0.37). See Figure 1c.

Repeated measures ANOVAs found no significant differences in anxiety scores (see Figure 1b), *F*(8, 8798.34) = 0.74, *p* = 0.67, or wellbeing scores (see Figure 1d) across time, *F*(8, 8833.22 = 1.19, *p* = 0.30).

## 3 Discussion

The present research used cricket to investigate the temporal component of mental health within sport. In doing so, the research has evidenced the importance of monitoring and supporting athletes' mental health year‐round and highlighted that discrete periods within a sporting cycle differentially contribute to mental health.

### 3.1 The Temporal Component of Mental Health

We monitored depression symptoms, alcohol misuse, anxiety symptoms, and wellbeing over three seasons. The rate of depression symptoms experienced and alcohol misuse significantly fluctuated across time; anxiety symptoms experienced and wellbeing remained stable across time. Specifically, PHQ scores rose from preseason to mid‐season, before then falling during the off‐season (see Figure 1a). Importantly, across the three seasons, there were no significant differences across the preseason levels of depression symptomology, indicating that although depression symptoms increased during the season, the off‐season and preseason served as a reset with depression symptom levels returning to their preseason baseline after the end of the season. This increase in the experience of depression symptoms during the season, across three seasons, suggests that the competition phase within a sporting cycle contributes to elevated symptoms of depression. Notably, based on the number of people meeting clinical thresholds (Table 1), these changes in PHQ‐9 scores appear to be subclinical. Regardless, the reset to lower depression levels during the off‐season and preseason suggests that time away from competition appears necessary to manage symptoms of depression over an athletic cycle, with the reset period likely affording athletes the capacity for mental rest [40]. Consequently, the off‐season can be deemed an important recharge period, and sports that continue to add fixtures to already congested calendars should consider the impact that this could have on athlete mental health.

For alcohol misuse, the off‐season was the period within the cricketing cycle where rates were highest. However, contrary to the accepted notion that alcohol consumption negatively influences mental health [41, 42], these spikes in alcohol consumption do not appear to be associated with increases in depression or anxiety, or decreases in wellbeing. Regardless of any relationship with our other markers of mental health, acknowledging that rates of alcohol consumption varied in relation to the sporting cycle highlights when alcohol‐related support is most likely required. Increases in alcohol misuse during the period within the athletic cycle away from the sporting environment also have relevance to research and practice concerning engagement in maladaptive behaviors upon retirement from sport.

The stability of anxiety symptoms and wellbeing throughout the 12‐month cycle is also of interest when compared to recent investigations into cricketer mental health. Research has established that wellbeing is tied to form during the playing season [14] and that coming to the end of a contract can be anxiety inducing [15]. While there is a time component in both instances (form derives from playing and thus can only be a factor during the playing season and contracts typically end at the end of the playing season), there also appears to be additional factors that influence the relationship between a discrete period in the sporting cycle and a mental health marker. Consequently, future research considering the temporal nature of athlete mental health would benefit from considering moderating and/or mediating variables as well as taking a qualitative approach to explore the temporal nature of athlete mental health.

### 3.2 Limitations

Whilst our use of MI in many ways is a strength of the research because it enabled longitudinal analysis of athlete mental health, having to use MI comes with limitations. For example, we are not able to report effect sizes for the ANOVA's. Additionally, our methods limited our capacity to add in controls such as age because of how we had to impute the data and conduct the analysis.

## 4 Conclusion

This research is the most comprehensive assessment of mental health in cricket to date and underscores the importance of longitudinal mental health monitoring. The significant fluctuation of mental health markers across a sporting cycle highlights the need for contextual awareness when conducting research and designing interventions. Additionally, the importance of time away from competition for mental health should be regarded as an important consideration for entities adding fixtures and lengthening playing seasons in all sports.

### 4.1 Perspective

The present study has demonstrated the importance of taking the next step in research regarding the monitoring of athlete mental health. Understanding of athlete mental health has progressed rapidly in the last decade, and a core component of said research has been the cross‐sectional monitoring of key elements of mental health. However, if core components of mental health differ within an athletic cycle, then to understand athlete mental health, data needs to be collected at multiple time points across an athlete's competitive cycle. Additionally, from an applied perspective, mental health monitoring and intervention needs to account for fluctuating mental health in relation to time‐specific factors. Knowledge that mental health changes within an athletic cycle now provides researchers and applied practitioners with a foundation for exploring the factors contributing to athlete mental health at the discrete points within the sporting cycle.

## Ethics Statement

Bangor University School of Sport and Exercise Science Ethics Committee approved the study.

## Consent

Consent was obtained via an opt‐in function built into the online survey. Prior to the dissemination of the survey, participants received a digital copy of the information sheet and the consent statement allowing them to ask questions and opt out of the study prior to survey dissemination.

## Conflicts of Interest

The authors declare no conflicts of interest.

## Supporting information


**Appendix S1:** sms70125‐sup‐0001‐AppendixS1.docx.

**Appendix S2:** sms70125‐sup‐0002‐AppendixS2.docx.

## Data Availability

The data that support the findings of this study are available on request from the corresponding author. The data are not publicly available due to privacy or ethical restrictions.
