# Supplementary material for: A Three‐Year Longitudinal Study of Athlete Mental Health: A Cricket Case Study
Source: Scand J Med Sci Sports. 2025 Aug 28;35(9):e70125. doi: 10.1111/sms.70125 (PMC12392049; doi:10.1111/sms.70125)
Supplement: Supplementary file 1 — Appendix S1: sms70125‐sup‐0001‐AppendixS1.docx. [file SMS-35-e70125-s002.docx]

**Supplementary Material 1 – Referral pathway**

Each data collection in this study also provided an opportunity to identify and offer support to individuals potentially in need of mental health-related support. Those aged 18 or older meeting the clinical threshold on the PHQ-9 or GAD-7 or endorsing item 9 on the PHQ-9 in any way were invited to be referred. For those under 18 meeting clinical thresholds, referral was automatic. For referrals, the survey sent an automatic email stating the reason for referral and the participant’s mental health code to the ECB employee who had created and held each player’s unique mental health code. That ECB employee then identified the person using the code and referred the player via their chosen pathway. PCA referrals went to the PCA’s Director of Member Services and ECB referrals went to the ECB’s Chief Medical Officer. Criteria and protocol for making a referral was devised by a governance group consisting of medical personnel and mental health professionals from the ECB and PCA.
