# Supplementary material for: A Three‐Year Longitudinal Study of Athlete Mental Health: A Cricket Case Study
Source: Scand J Med Sci Sports. 2025 Aug 28;35(9):e70125. doi: 10.1111/sms.70125 (PMC12392049; doi:10.1111/sms.70125)
Supplement: Supplementary file 2 — Appendix S2: sms70125‐sup‐0002‐AppendixS2.docx. [file SMS-35-e70125-s001.docx]

**Supplementary Material**

**Repeated Measures Analysis of Variance Example R Code**

#packages

library(mice)

library(mitml)

library(miceadds)

library(lme4)

library(lattice)

#Asigning each individual an ID (cluster variables need to be continous)

data <- tibble::rowid_to_column(temp_name_for_test, "ID")

# Getting just depression

JustDepression <- dplyr::select(data, "ID", "AgeS1", DEPRESSIONP1:DEPRESSIONO3)

#changing data from wide to long (required for repeated measure Anova)

depression_long <- JustDepression %>% pivot_longer(cols = c('DEPRESSIONP1', 'DEPRESSIONM1', 'DEPRESSIONO1','DEPRESSIONP2','DEPRESSIONM2','DEPRESSIONO2', 'DEPRESSIONP3', 'DEPRESSIONM3', 'DEPRESSIONO3'),

names_to = 'Time',

values_to = 'Score')

# run MI (for starting solution)

ini <- mice(data = depression_long, maxit = 0)

#Change predictor matrix

# define the cluster variable (code as '-2' in predictor matrix)

pred <- ini$pred

pred["Score", "ID"] <- -2

#make time a variable in predictor matrix

pred["Score", "Time"] <- 1

# run MI (for real)

imp2 <- mice(data = depression_long, pred = pred, method = "2l.pmm", m = 100, maxit = 20)

summary(imp2)

# create a list of completed data sets

implist2 <- mids2mitml.list(imp2)

library(lme4)

# fit the ANOVA model (the '(1|id)' accounts for nested structure of repeated measure data)

fit2 <- with(implist2, lmer(Score ~ 1 + Time + (1|ID)))

testEstimates(fit2, extra.pars = TRUE)

# pool the parameter estimates - WHERE WE TEST THE NULL HYPOTHESIS

fit2.reduced <- with(implist2, lmer(Score ~ 1 + (1|ID)))

testModels(fit2, fit2.reduced, method = "D1")

#POST-HOC

library(multcomp)

# perform pairwise comparisons (Tukey)

fit2.pairwise <- lapply(fit2, glht, linfct = mcp(Time = "Tukey"))

# convert to "mitml.result" and pool the parameter estimates

fit2.pairwise <- as.mitml.result(fit2.pairwise)

testEstimates(fit2.pairwise)
